# The effect of allyl isothiocyanate on chondrocyte phenotype is matrix stiffness-dependent: Possible involvement of TRPA1 activation

**DOI:** 10.3389/fmolb.2023.1112653

**Published:** 2023-03-16

**Authors:** Hui Che, Zhiqiang Shao, Jiangchen Ding, Hua Gao, Xiangyu Liu, Hailong Chen, Shuangyu Cai, Jiaying Ge, Chengqiang Wang, Jun Wu, Yuefeng Hao

**Affiliations:** ^1^ Orthopedics and Sports Medicine Center, The Affiliated Suzhou Hospital of Nanjing Medical University, Suzhou Municipal Hospital, Gusu School, Nanjing Medical University, Suzhou, China; ^2^ The Research Center for Bone and Stem Cells, Department of Anatomy, Histology and Embryology, Nanjing Medical University, Nanjing, Jiangsu, China

**Keywords:** TRPA1, chondrocyte, osteoarthritis, MRTF-A, pSOX9, inflammation, F-actin cytoskeleton, matrix stiffness

## Abstract

Osteoarthritis (OA) is a chronic joint disease with increasing prevalence. Chondrocytes (CHs) are highly differentiated end-stage cells with a secretory phenotype that keeps the extracellular matrix (ECM) balanced and the cartilage environment stable. Osteoarthritis dedifferentiation causes cartilage matrix breakdown, accounting for one of the key pathogenesis of osteoarthritis. Recently, the activation of transient receptor potential ankyrin 1 (TRPA1) was claimed to be a risk factor in osteoarthritis by causing inflammation and extracellular matrix degradation. However, the underlying mechanism is still unknown. Due to its mechanosensitive property, we speculated that the role of TRPA1 activation during osteoarthritis is matrix stiffness-dependent. In this study, we cultured the chondrocytes from patients with osteoarthritis on stiff vs. soft substrates, treated them with allyl isothiocyanate (AITC), a transient receptor potential ankyrin 1 agonist, and compared the chondrogenic phenotype, containing cell shape, F-actin cytoskeleton, vinculin, synthesized collagen profiles and their transcriptional regulatory factor, and inflammation-related interleukins. The data suggest that allyl isothiocyanate treatment activates transient receptor potential ankyrin 1 and results in both positive and harmful effects on chondrocytes. In addition, a softer matrix could help enhance the positive effects and alleviate the harmful ones. Thus, the effect of allyl isothiocyanate on chondrocytes is conditionally controllable, which could be associated with transient receptor potential ankyrin 1 activation, and is a promising strategy for osteoarthritis treatment.

## Introduction

Articular cartilage (AC) is a layer of connective tissue that covers the surface of a joint and plays a role in lubrication, load distribution, and shock absorption during joint movement. The AC tissue is mainly nourished by the joint fluid but lacks blood vessels and nerves. Trauma, natural degeneration, inflammation, and other factors can lead to progressive degradation of AC and, eventually, cause osteoarthritis (OA) (Quicke et al., 2022). OA is one of the most common chronic degenerative diseases characterized by progressive degradation of AC, thickening of subchondral bone, and formation of osteophyte, resulting in loss of joint mobility and function. It affects millions of individuals worldwide, yet treatment choices are sometimes restricted (Zhou et al., 2022). As it is normally inflammation-related, the normal clinical treatment options for OA (Meyer et al., 2021) are hyaluronic acid non-steroidal anti-inflammatory drugs and operations, which can relieve painful symptoms, improve joint mobility and delay disease progression but have significant side effects. Therefore, research into potential targets for the treatment or prevention of OA is particularly important (Lesage et al., 2022).

Chondrocyte (CH), as the only cell type in AC, plays an important role in regulating the synthesis and degradation of the extracellular matrix (ECM) and maintaining cartilage matrix homeostasis, and its dysfunction or dedifferentiation is a major pathological factor in OA (Meyer et al., 2021; Pei et al., 2022; Segarra-Queralt et al., 2022). Transient receptor potential (TRP) channels are cation-selective transmembrane receptors acting as physiological sensors for physical and chemical stimuli, containing PH, temperature, mechanical stress, and osmolarity, and regulating cellular responses to these stimuli. TRP channel dysfunction in CHs may be a causative factor in inflammation, mechanical and osmotic stress, joint pain, and final disorders, and is therefore a possible target for OA treatment (Krupkova et al., 2017).

In mammals, the TRP channels are classified into six subfamilies based on sequence homology and topological differences: TRPA, TRPC, TRPM, TRPV, TRPP, and TRPML. TRPA1 is the only one that mediates pain and nociceptive sensitization in neuronal and non-neuronal cells (Meents et al., 2019). In terms of the study of OA, a rat model in 2010 (McGaraughty et al., 2010) was the first to report that TRPA1 mediates the transmission of low-intensity mechanical stimulation. Recently, TRPA1 has been identified to be functionally expressed in primary human OA CHs for its role in modulating synovial inflammation and cartilage deterioration (Moilanen et al., 2015b; Horváth et al., 2016), which has been attributed mostly to a TRPA1-induced imbalance in the synthesis of catabolic, anabolic, and inflammatory mediators (Nummenmaa et al., 2016; Andrei et al., 2017). However, to date, minimal emphasis has been dedicated to defining the relationship between TRPA1 and CHs.

TRPA1 activation has been shown to cause an influx of cations, particularly Ca^2+^, acting as a trigger of cytokine secretion and gene transcription to mediate cellular metabolism and the production of proinflammatory neuropeptides such as substance P, calcitonin gene-related peptide, and neurokinin (Bautista et al., 2013). Its activation was associated with the stimulation of inflammatory factors, such as TNF-α and IL-1β (Fernandes et al., 2011; Hatano et al., 2012). Furthermore, the suppression of TRPA1 by its antagonist HC-030031 or TRPA1 gene knockout ameliorates the chondrogenic phenotype of CH and prevents OA (Moilanen et al., 2015a; Moilanen et al., 2015b; Nummenmaa et al., 2016).

Based on the N-terminal ankyrin repeat domain, TRPA1 was also reported as an intrinsically mechanosensitive ion channel involved in the transduction of noxious cold and mechanical stimuli in hair cells and neurons (Story and Gereau, 2006; Eid et al., 2008; Barrière et al., 2013). In addition, the degeneration of the functional collagen II and aggrecan and the production of fibrous collagen I pathologically altered ECM matrix profiles, causing an increasing mechanical stiffness of AC (Hodgkinson et al., 2022). Therefore, we wondered whether the effect of TRPA1 activation on CHs is associated with the mechanical environment.

Allyl isothiocyanate (AITC), a component in mustard oil and widely existing in cruciferous vegetables, is one of the natural constituents of isothiocyanates. It has an electrophilic group that selectively binds to the cysteine residues of TRPA1, which has been frequently used as the agonist of TRPA1 in OA research (Nummenmaa et al., 2016). In addition, AITC supplementation has shown an inhibition of mechanical allodynia and protection of nociception and functional disability associated with OA pain (Batallé et al., 2019). Therefore, we speculated that AITC treatment to OA is also mechanically related, potentially involving TRPA1 activation.

In this study, we isolated mild dedifferentiated CHs from patients with OA and cultured them on a stiff (plastic plate) or soft substrate (polyacrylic acid (PAA) hydrogel). Meanwhile, we treated CHs with AITC and proved the activation of the TRPA1 channel. After 6 days of treatment, collagen II and I, their regulator myocardin-related transcription factor A (MRTF-A) and phosphorylated-Sox9 (pSox9), cell shape descriptors, F-actin cytoskeleton, vinculin, and inflammatory-related interleukin-6/10 were analyzed to reveal the mechanism underlying the effects of AITC treatment on CH phenotype. The results clarify that these effects could be matrix stiffness- and TRPA1 activation-related and helps us understand the role of TRPA1 during the process of OA and provide new insights into AITC treatment for OA intervention.

## Materials and methods

### Patient cartilage collection

We collected 10 fresh human knee cartilage samples from five patients with OA undergoing total knee arthroplasty and five knee cartilage defect patients (no OA history) undergoing fracture internal fixation in our hospital. The five samples of knee fracture were used as control. Informed consent from the patients was obtained before the surgery. This project was approved by the Ethics Committee of The Affiliated Suzhou Hospital of Nanjing Medical University (registered number 2022-594).

### Immunohistochemical staining (IHC)

Human cartilage tissue was fixed in 4% paraformaldehyde solution (Beyotime, China), embedded in paraffin, and sectioned into 5-mm-thick slices for the following use. For antigen retrieval, the slices were treated with boiling sodium citrate (10 mM). Then, the slices were blocked with 10% bovine serum albumin (BSA) and incubated with anti-TRPA1 (ab62053, Abcam, United States) overnight at 4 °C. The next day, the slices were treated with goat anti-rabbit IgG H&L (HRP) (ab205718, Abcam, United States) and incubated with VECTASTAIN Elite ABC reagent (Fisher Scientific, United States) for 30 min and 3,3-diaminobenzidine and hematoxylin for counterstaining.

### Western blot (WB)

The nuclear protein of CHs was extracted by using the CelLytic NuCLEAR kit (Sigma-Aldrich, United States) according to the instruction manual. WB was performed as previously described (Che et al., 2020). All the blots were incubated with primary antibodies: MRTF (Abcam, United States), pSOX9 (Abcam, United States), collagen Ⅰ and Ⅱ (Abcam, United States), and β-actin (Proteintech, United States).

### Enzyme-linked immunosorbent assay (ELISA)

Cell lysis samples were obtained from cells collected after treatment in each group. The levels of IL-6 and IL-10 in CHs were determined using an ELISA kit according to the instruction manual (KeyGen, Nanjing, China).

### Quantitative real-time polymerase chain reaction (qRT-PCR)

Total RNA from the cartilage tissue or CHs was extracted using TRIzol reagent (Beyotime, China) and was then used for cDNA reverse transcription by PrimeScript RT Master Mix (Thermo, United States). Sequencing methods such as qRT-PCR and the 2^−ΔΔCt^ method were performed to determine the TRPA1 gene expression, which had been normalized by GAPDH. The applied TRPA1 primer was Forward 5′-TCCTCTCCATCTGGCAGCAAAG-3′and reverse 5′-GGA​CGC​ATG​ATG​CAA​AGC​TGT​C-3’ and GAPDH primer was Forward 5′-GTCTCCTCTGACTTCAACAGCG-3′and reverse 5′- ACC​ACC​CTG​TTG​CTG​TAG​CCA​A-3’.

### PAA hydrogel preparation

The PAA hydrogel was polymerized by mixing ddH_2_O, 10×PBS, 2% bis-acrylamide, 30% acrylamide, APS, and TEMED (Carl Roth GmbH and Bio-Rad, Germany). Before cell culturing, the surface of the hydrogel was incubated with fibronectin for cell adhering.

### CH isolation and culture

The cartilage tissue was fragmented using a scalpel and then was shaken (250 rpm) in collagenase Ⅱ at 37 °C with 95% humidity overnight. The next day, the cell digestate was filtered using a strainer and centrifuged at 1000 rpm for 10 min to obtain the CH pellets. CHs were resuspended in expansion media (DMEM+10% FBS+ 2% penicillin/streptomycin, ThermoFisher Scientific, United States) until the first passage for experimental use. CHs were divided into two groups: cultured on a 24-well plastic culture plate or cultured on PAA hydrogel. Each group was subdivided into three subgroups: 1-day culture without AITC treatment, 6-day culture without AITC treatment, and 6-day culture with AITC (20 μM, Sigma, United States) treatment. All the media were changed every 3 days.

#### Ca^2+^-influx measurements

Ca^2+^ influx was measured to present the TRPA1 activity as described previously (Moilanen et al., 2016). Briefly, the cells were incubated with the working solution, containing fluo-3-acetoxymethyl ester (4 μM, Sigma, United States) + HEPES (pH 7.2, 25 mM) + BSA (1 mg/mL) + probenecid (2.5 mM) + Pluronic F-127 (0.08%, all from Millipore Sigma) for 30 min at room temperature. Then, the intracellular-free Ca^2+^ levels were assessed by using a VICTOR3 1420 multilabel counter (Perkin Elmer, United States) at excitation/emission wavelengths of 485/535 nm. The Ca^2+^ influx in the CHs of the abovementioned six groups was first measured for 45 s and then continued for 30 s with adding the control ionophore compound ionomycin (1 μM, Millipore Sigma).

#### Immunofluorescence (IF)

After harvesting, CHs were fixed by 4% formaldehyde for 15 min, incubated in 0.1% Triton X (Beyotime, China) for 15 min, and blocked by 2% BSA (Sigma, United States) for 1 h. Then, the cells were incubated with anti-pSOX9, anti-MRTF, anti-collagen Ⅰ and Ⅱ, anti-IL-6 and 10, and anti-vinculin overnight at 4 °C. Then, the cells were incubated with DyLight 488 or 647 goat anti-rabbit IgG antibody for 1 h in the dark. In addition, nuclei were counterstained with DAPI and the cytoskeleton was stained with phalloidin. All the antibodies were purchased from Abcam. The images were taken by a fluorescence microscope (Zeiss, Germany) and analyzed using ImageJ software (National Institutes of Health, United States).

### Statistical analysis

All data were analyzed and graphs were generated by GraphPad Prism 9 software. All data were analyzed for normality (Kolmogorov–Smirnov test) and are presented as a dot plot with mean ± standard deviation (SD) or boxplots indicating the minimum, first quartile, median, third quartile, and maximum of the data. Student’s t-test was used for comparing the differences between the two groups, and ANOVA on ranks was applied for pairwise multiple comparisons between more than two groups. The differences were considered statistically significant at *p* < 0.05.

## Result

### TRPA1 was overexpressed in OA cartilage

**FIGURE 1:**
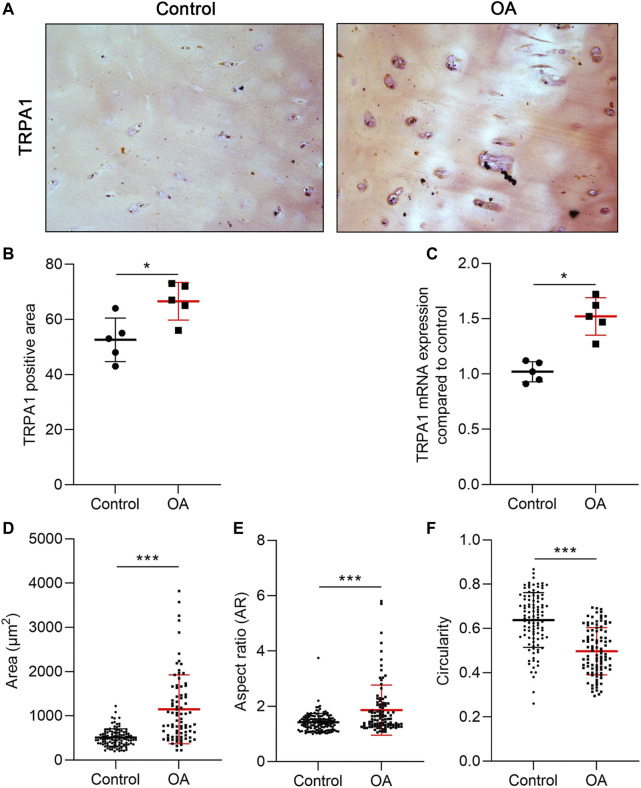
TRPA1 was overexpressed in OA cartilage. **(A)** IHC staining of TRPA1; **(B)** the quantitative analysis of the TRPA1-positive area; **(C)** RT-PCR analysis of the TRPA1 mRNA expression compared to the control; and **(D–F)** area, AR, and circularity of the CHs in the control and OA tissue. Results are expressed as dot plots with mean +SD (*n* = 5 samples in the IHC and PCR analysis; *n* = 100–120 cells in the cell shape measurement). An unpaired *t*-test was used in the statistical analysis. The differences are considered statistically significant at *p < 0.05 and ***p < 0.001.

The TRPA1 gene is expressed in OA cartilage, and it was upregulated in the IL-1β-treated CHs (Nummenmaa et al., 2016). To confirm the different expression levels of TRPA1 between the normal and OA cartilage, we collected the cartilage with no OA symptoms or visible degeneration from the fracture patients as normal control and the cartilage from OA patients as the OA group. We analyzed the TRPA1 expression using IHC and RT-PCR methods. Compared to the control group, the population of TRPA1-positive CHs in OA tissue was slightly increased (Figures 1A, B). Additionally, the mRNA level of TRPA1 was higher in the OA group than in the control (Figure 1C), indicating the overexpressed TRPA1 protein or mRNA could be a biomarker of OA. Moreover, we analyzed the shape of CHs in the cartilage tissue and found that the cell size and aspect ratio (calculated by the long axis divided by the short axis of the cell) were increased in OA conditions compared to the control, and the circularity was decreased in OA (Figures 1D–F). Therefore, CHs in OA present a fibrotic appearance and a hypertrophic shape with an increased TRPA1 level.

### AITC activated TRPA1 but did not promote its expression

To clarify whether AITC treatment influences OA development and is associated with TRPA1 activation and dependent on matrix stiffness, we treated the CHs with AITC on substrates of different stiffness, namely, the normal plastic culture plate and a self-made PAA hydrogel surface. To confirm the functional activation of the TRPA1 channel after AITC stimuli, we measured the cellular Ca^2+^-influx, with an increased influx presenting an activated condition.

**FIGURE 2:**
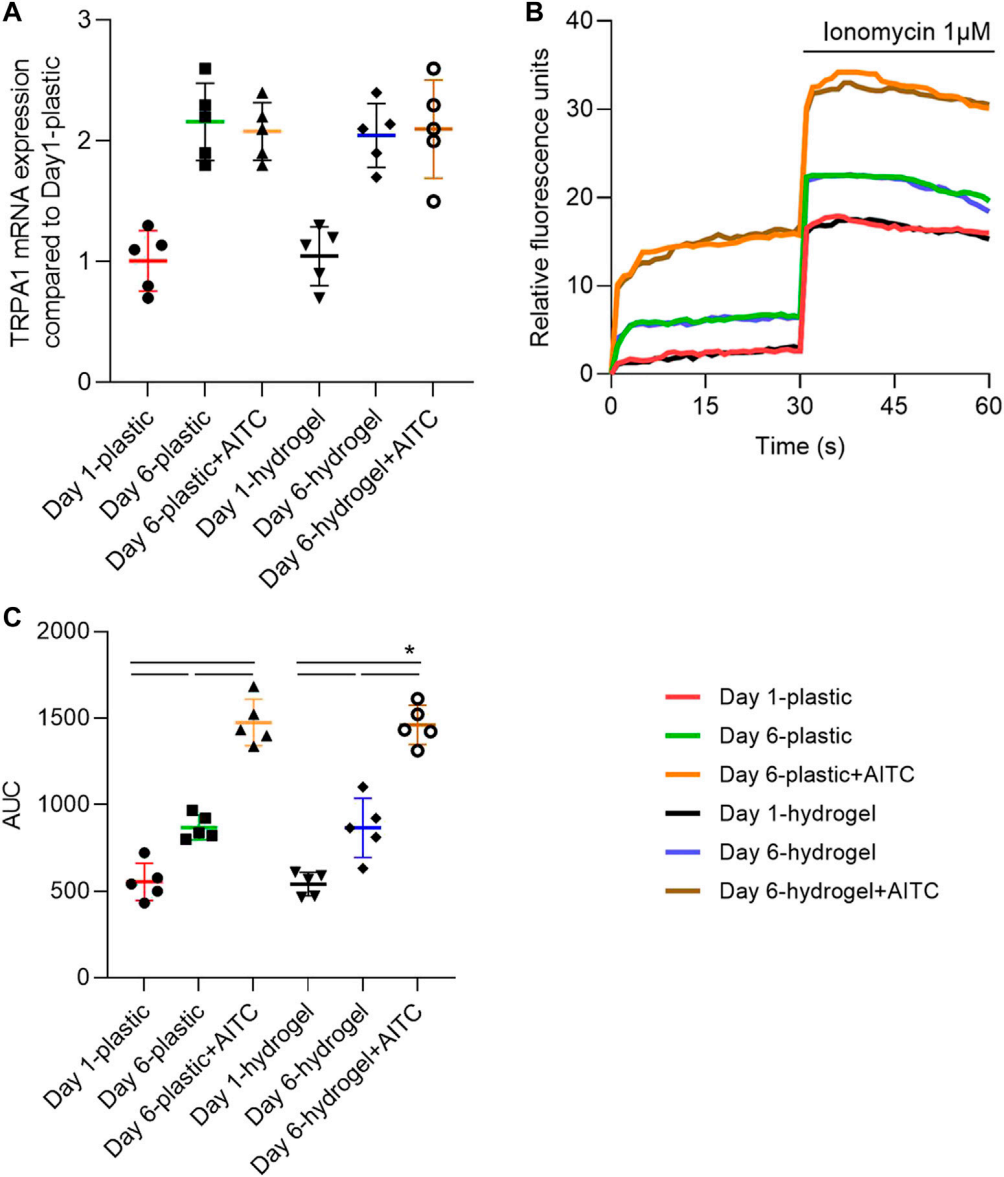
TRPA1 was functionally activated by AITC stimuli. CHs were divided into two groups: one was cultured on plastic and the other was cultured on hydrogel. Each group was then sub-grouped into three: 1-day culture, 6-day culture, and 6-day culture with AITC treatment. **(A)** TRPA1 mRNA expression was determined by RT-PCR; **(B)** one representative experiment illustrated the activity of the TRPA1 channel by showing the Ca^2+^ influx; **(C)** area under the curve (AUC) from 0 to 30 s was calculated from measurements of CHs from three donors (each with three repeats). Results are expressed as dot plots with mean +SD (*n* = 5 samples). Repeated measures ANOVA followed by Dunnett’s post-test was used in the statistical analysis. The differences are considered statistically significant at *p* < 0.05.

We first verified whether the AITC could upregulate TRPA1 expression using RT-PCR. From days 1 to 6, the TRPA1 level was significantly increased on both the plastic and hydrogel surfaces, which was not further increased by adding AITC. In addition, the TRPA1 expression on the plastic surface was not significantly different from that on the hydrogel surface (Figure 2A). Thus, TRPA1 expression was time-dependent with the degeneration of CHs but not affected by the agonist or matrix stiffness. To illustrate the TRPA1 channel function, the fluorescence units every second was recorded and one representative Ca^2+^-influx curve of each group is presented in Figure 2B. Meanwhile, we also applied the ionophore compound ionomycin in the last 30 s to amplify and increasingly evidentiate the Ca^2+^-influx reaction. The result indicated a more robust Ca^2+^ influx on day 6 than on day 1, which can be further increased by AITC. After adding ionomycin, the Ca^2+^ influx was immediately increased and then slowly declined, the curves of AITC treatment were still the highest, and the curves of day 1 were the lowest. Interestingly, it seems that the difference in stiffness did not change the Ca^2+^ influx of CHs.

To quantify the Ca^2+^ influx, we calculated the area under the curve (AUC) of each condition in the first 30 s (Figure 2C). We found that Ca^2+^ influx was increased from day 1 to day 6 and continuously increased with the treatment of AITC on both plastic and hydrogel. However, the culturing on hydrogel did not cause more Ca^2+^ influx compared to the plastic surface. Therefore, these data suggested that long-time culturing caused more functional TRPA1 expression, AITC treatment only activated the TRPA1 activity but did not induce increased expression, and the soft substrate did not influence either the expression or the activity of TRPA1.

### AITC treatment regulated the shape and F-actin cytoskeleton of CHs

**FIGURE 3:**
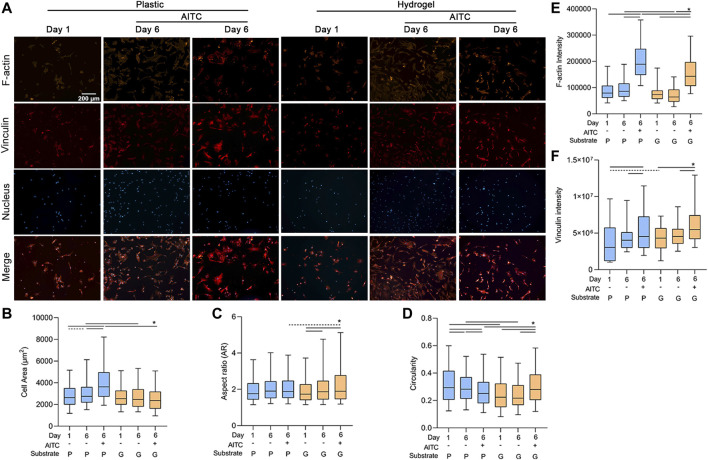
TRPA1 activation regulated the shape and F-actin cytoskeleton of CHs. CHs were divided into two groups: one was cultured on plastic and the other was cultured on hydrogel. Each group was then sub-grouped into three: 1-day culture, 6-day culture, and 6-day culture with AITC treatment. **(A)** Representative images of F-actin, vinculin, and nucleus staining of CHs, and the quantitative analysis of **(B)** cell area, **(C)** AR, **(D)** circularity, **(E)** F-actin intensity, and **(F)** vinculin intensity. Results are expressed using the boxplots (*n* = 80–200 cells). Repeated measures ANOVA followed by Dunnett’s post-test was used in the statistical analysis, and a *t*-test was also applied when the difference was not significant with the ANOVA test. The differences are considered statistically significant at *p* < 0.05. (P: plastic, G: hydrogel; line: line on the top of the boxes points to the compared objects with ANOVA, dot line: test with *t*-test).

Long-term monolayer culture causes CH natural dedifferentiation. Changing cell morphology is a typical characteristic of CH dedifferentiation, which switches from a small and round shape to a swollen and fibrotic shape. We measured the cell area, AR, and circularity of CHs using the staining of the F-actin cytoskeleton (Figure 3A). The area of CHs increased from day 1 to 6 on plastic, and AITC treatment induced the CHs to grow to a much larger size compared to the non-treated group. However, the cell area was stable on day 6 when culturing on hydrogel and even with the AITC stimuli. Compared to plastic, culturing on hydrogel prevented the enlargement of the cell area on day 6 with AITC supplementation or without (Figure 3B). However, the 6-day culture and AITC treatment did not affect the AR of CHs on plastic, whereas the AR significantly increased from day 1 to day 6 and excessively increased under AITC treatment on the hydrogel (Figure 3C). In terms of cell circularity, 6-day culturing on plastic decreased the circularity and it was further reduced with the AITC supplement. On the hydrogel, the cell circularity did not change during the 6 days of culture and increased with the addition of AITC. Compared to plastic, growing on hydrogel prevented the reduction of circularity and increased it with AITC supplementation (Figure 3D). Therefore, a softer substrate maintained a small but irregular cell shape of CHs, and how AITC affected the cell shape was dependent on the matric stiffness.

As cell shape is affected by the cytoskeleton, we also analyzed the amount of F-actin in each group. On the plastic and hydrogel surfaces, the F-actin intensity was not changed after the 6-day culture, but AITC significantly promoted its expression. Comparing different stiffness, the F-actin intensity was lower on a soft material on day 6 irrespective of having AITC stimuli or not (Figure 3E). To clarify whether the increase of F-actin was associated with excessive vinculin expression, we also stained vinculin in the same cell and found that AITC could promote vinculin expression on both plastic and hydrogel (Figure 3F). Therefore, the AITC-caused F-actin upregulation may be related to increased vinculin expression.

### AITC treatment promoted collagen Ⅰ and Ⅱ synthesis

**FIGURE 4:**
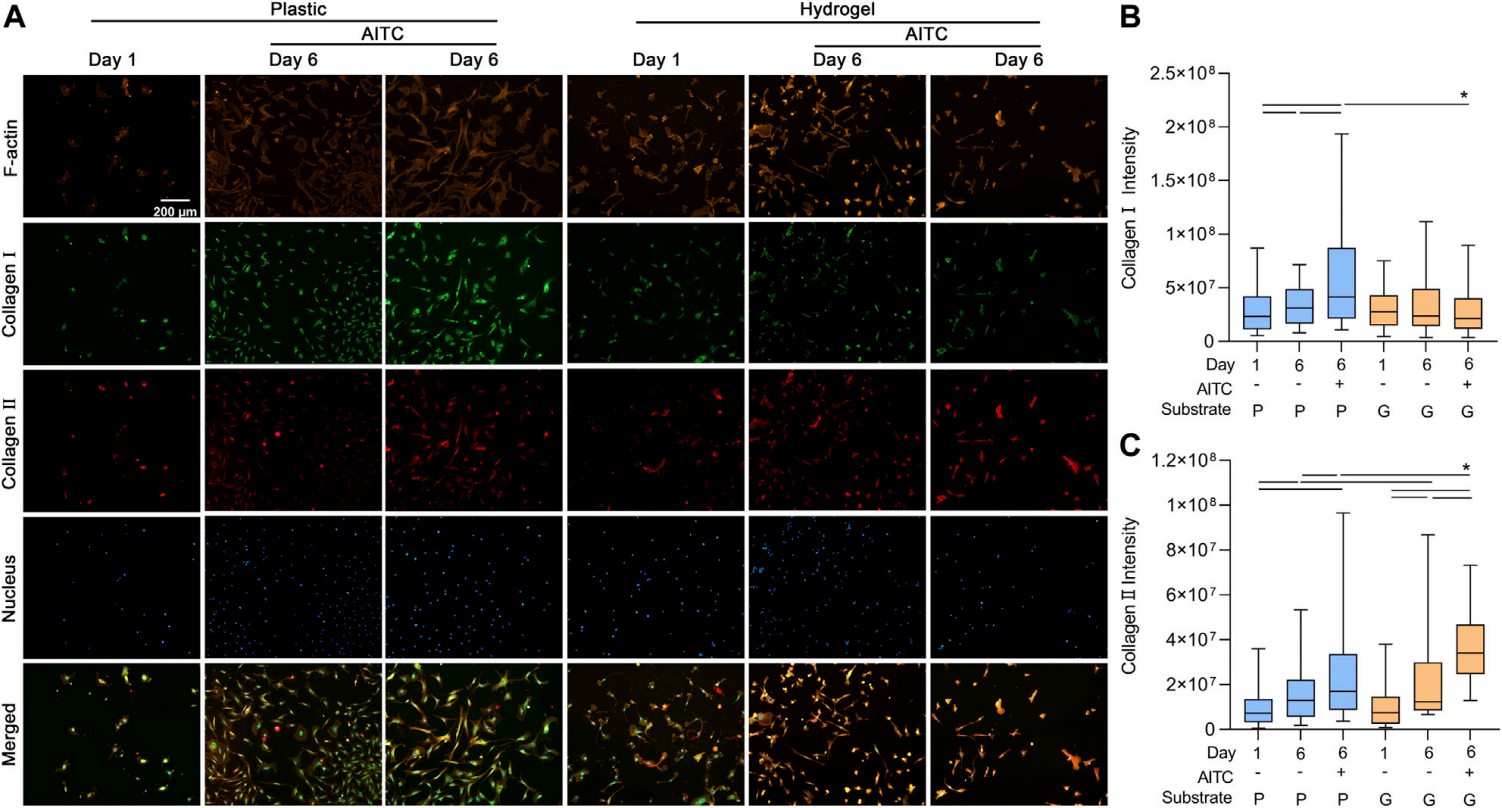
TRPA1 activation promoted collagen Ⅰ and Ⅱ synthesis. CHs were divided into two groups: one was cultured on plastic and the other was cultured on hydrogel. Each group was then sub-grouped into three: 1-day culture, 6-day culture, and 6-day culture with AITC treatment. **(A)** Representative images of F-actin, collagen Ⅰ/Ⅱ, and nucleus staining of CHs, and the quantitative analysis of **(B)** collagen Ⅰ intensity and **(C)** collagen Ⅱ intensity. Results are expressed using the box plots (*n* = 80–200 cells). Repeated measures ANOVA followed by Dunnett’s post-test was used in the statistical analysis. The differences are considered statistically significant at *p* < 0.05. (P: plastic, G: hydrogel; line: line on the top of the boxes points to the compared objects with ANOVA).

During the dedifferentiation of CHs, there was a switch regarding collagen synthesis with an increased collagen Ⅰ and decreased collagen Ⅱ expression. To clarify how AITC treatment affects the synthesis of collagen Ⅰ and Ⅱ, we used the IF method to analyze collagen Ⅰ and Ⅱ expression and also stained the F-actin to indicate the location of collagens in the same CH. With time, collagen Ⅰ and Ⅱ were accumulated in the cell, and some of them were secreted around the cell; thus, the IF staining showed not only the total amount of protein before the cell was fixed but also the last moment before death. As shown in Figure 4A, all the collagen Ⅰ and Ⅱ gathered around the nucleus because they were synthesized from the nucleus. On the plastic surface, the accumulation of collagen Ⅰ and collagen Ⅱ increased from day 1 to 6, and AITC triggered significant production of collagen Ⅰ and Ⅱ. On the hydrogel surface, the amount of collagen Ⅰ was neither increased over time nor influenced by AITC supplementation, but collagen Ⅱ production was increased from day 1 to 6 and enhanced by AITC supplementation. Compared to plastic, CHs growing on hydrogel produced more collagen Ⅱ with or without AITC treatment on day 6 and less collagen Ⅰ when treated with AITC. Therefore, a soft substrate prevented collagen Ⅰ but promoted collagen Ⅱ expression. On a stiff surface, AITC enhanced both collagen Ⅰ and Ⅱ synthesis while enhancing only collagen Ⅱ synthesis on a soft substrate (Figure 4B, C).

### AITC treatment promoted the pSOX9 and MRTF expression level

**FIGURE 5:**
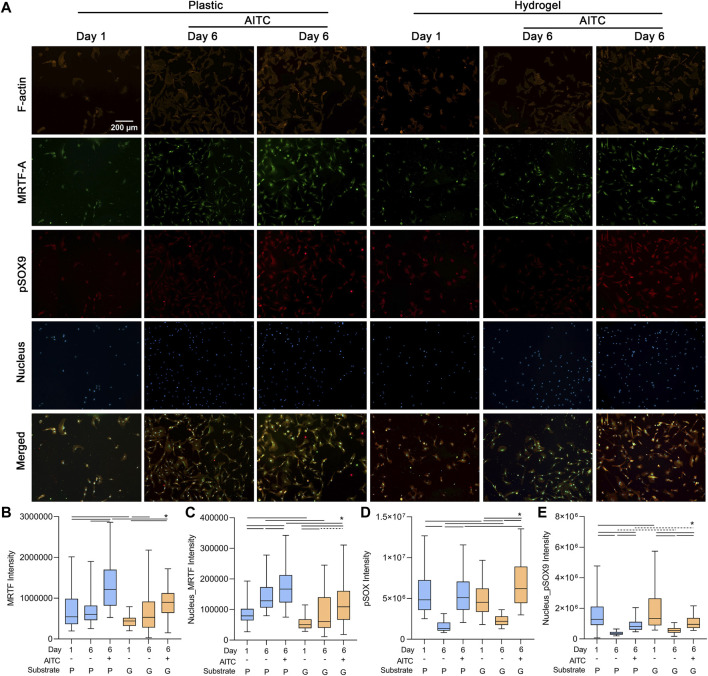
TRPA1 activation promoted pSOX9 and MRTF expression. CHs were divided into two groups: one was cultured on plastic and the other was cultured on hydrogel. Each group was then sub-grouped into three: 1-day culture, 6-day culture, and 6-day culture with AITC treatment. **(A)** Representative images of F-actin, MRTF-A, pSOX9, and nucleus staining of CHs, and the quantitative analysis of **(B)** total cellular MRTF-A, **(C)** nuclear MRTF-A, **(D)** total cellular pSOX9, and **(E)** nuclear pSOX9 intensity. Results are expressed using the boxplots (*n* = 80–200 cells). Repeated measures ANOVA followed by Dunnett’s post-test was used in the statistical analysis, and a *t*-test was also applied when the difference was not significant with the ANOVA test. The differences are considered statistically significant at *p* < 0.05. (P: plastic, G: hydrogel; line: line on the top of the boxes points to the compared objects with ANOVA, dot line: test with *t*-test).

As collagen Ⅰ is regulated by the transcriptional coactivator MRTF-A and collagen Ⅱ is mediated by transcriptional factor pSOX9, we separately analyzed the MRTF-A and pSOX9 levels in the total cell and nucleus to further clarify how TRPA1 activation regulated collagen expression. Additionally, we also stained F-actin to illustrate the location of MRTF-A and pSOX9 in CHs (Figure 5A).

On the plastic surface, the total cellular MRTF-A was not changed from days 1 to 6, but the nuclear MRTF-A was increased, indicating it transferred from the cytosol to the nucleus. AITC treatment could induce both cellular and nuclear MRTF-A expression. On the hydrogel surface, the cellular and nuclear MRTF-A were both increased from days 1 to 6, and AITC did not promote its total level but the nuclear level. The cellular and nuclear MRTF-A growing on the hydrogel were always lower than those on plastic, except in the day 6 group with no AITC treatment (Figures 5B, C). Moreover, the 6-day culture caused a significant loss of cellular and nuclear pSOX9 on both plastic and hydrogel. On the plastic, the AITC supplementation could prevent cellular loss and alleviate the nuclear loss. On the hydrogel, AITC could promote more cellular pSOX9 production than on day 1 and rescue some nuclear loss. Compared to growth on plastic, the cellular and nuclear pSOX9 growths were always higher on hydrogel, except for the group of cellular pSOX9 on day 1 (Figures 5D, E). Therefore, AITC treatment on a stiff substrate enhanced the production of both total cellular and nuclear MRTF-A and pSOX9. In contrast, AITC treatment on a soft substrate only upregulated cellular pSOX9 but not MRTF-A, and it led to less MRTF-A, but more pSOX9 nuclear translocation.

**FIGURE 6:**
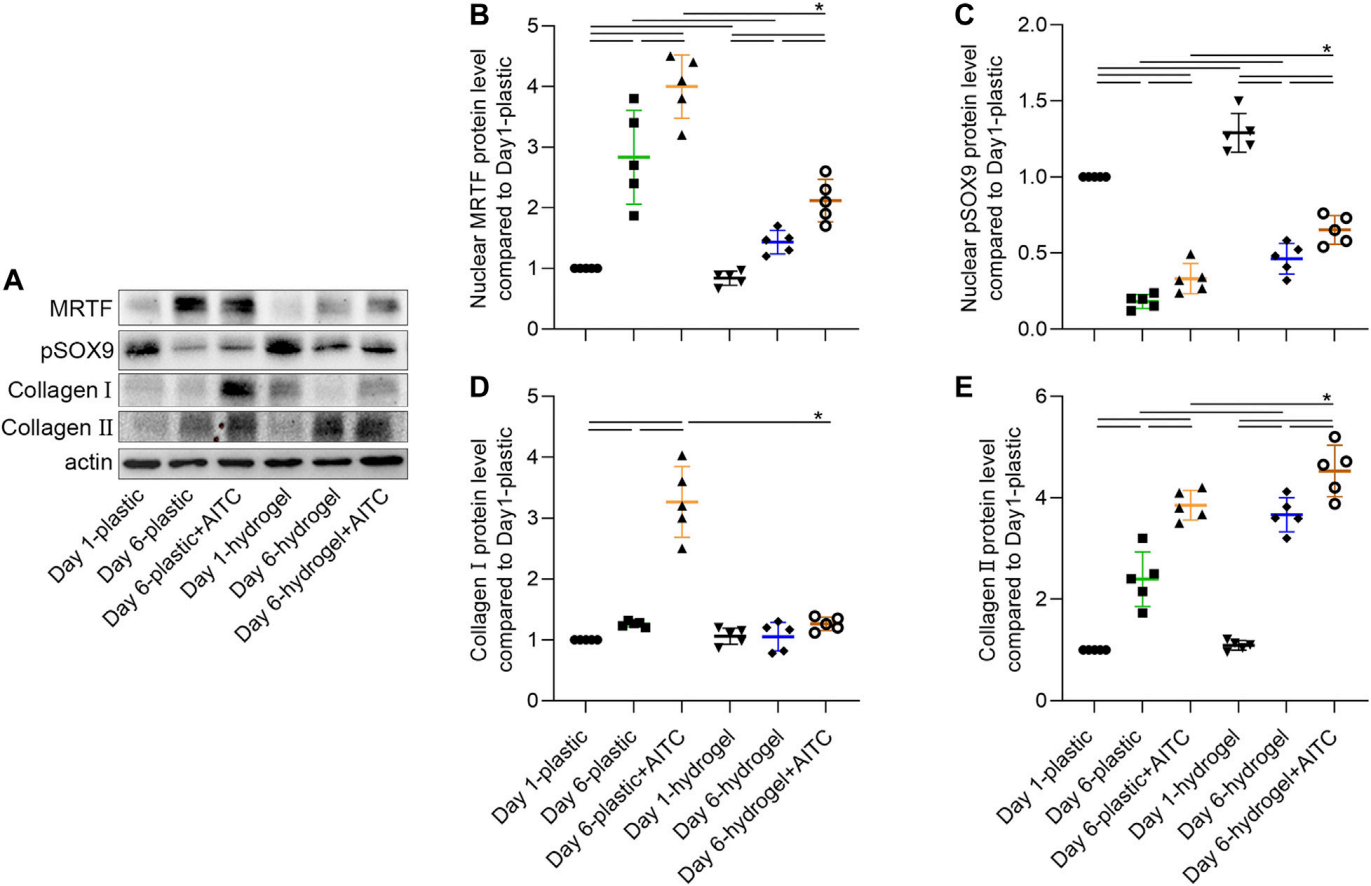
WB analysis of nuclear MRTF-A, pSOX9, and collagen Ⅰ/Ⅱ expression. CHs were divided into two groups, one was cultured on plastic and the other was cultured on hydrogel. Each group was then sub-grouped into three: 1-day culture, 6-day culture, and 6-day culture with AITC treatment. **(A)** Representative images of the blots of each target, and the quantitative analysis of **(B)** nuclear MRTF-A, **(C)** nuclear pSOX9, and **(D)** collagen Ⅰ and **(E)** collagen Ⅱ expression. Results are expressed using the boxplots (*n* = 5 samples). Repeated measures ANOVA followed by Dunnett’s post-test was used in the statistical analysis. The differences are considered statistically significant at *p* < 0.05. (P: plastic, G: hydrogel).

The nuclear translocation of MRTF-A and pSOX9 and the associated collagen Ⅰ and Ⅱ expression were also verified by the WB method (Figures 6A, B). As the previously mentioned trends indicate, the increased nuclear MRTF-A level was time- and AITC-dependent, of which a much higher expression was observed on a stiff substrate than on a soft one; the nuclear pSOX9 level was decreased over time, but a soft surface and AITC treatment would rescue the loss; a soft substrate was effective to prevent collagen Ⅰ secretion even with the existence of AITC; the collagen Ⅱ level was increased over time and by AITC treatment, of which a much higher expression was observed on a soft substrate than on a stiff one. Thus, AITC treatment in a soft substrate caused more pSOX9 to enter the nucleus and more collagen Ⅱ synthesis, which is beneficial for maintaining the chondrogenic phenotype.

### AITC treatment regulated the inflammation of CHs

**FIGURE 7:**
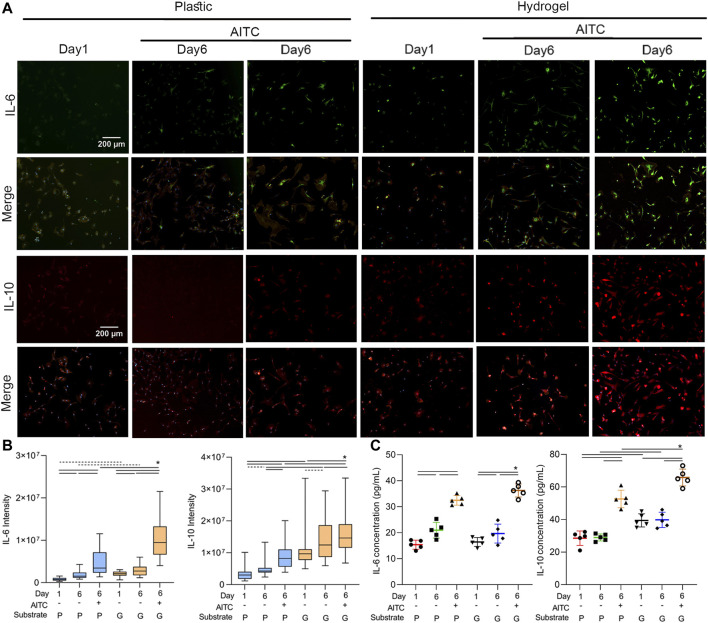
TRPA1 activation regulated the inflammation of CHs. CHs were divided into two groups: one was cultured on plastic and the other was cultured on hydrogel. Each group was then sub-grouped into three: 1-day culture, 6-day culture, and 6-day culture with AITC treatment. **(A)** Representative images of IL-6 or IL-10 and merged with F-actin and nucleus staining of CHs, and the quantitative analysis of **(B)** IL-6 and IL-10 intensity; **(C)** ELISA measurement of cellular IL-6 and IL-10 levels. Results are expressed using the boxplots (*n* = 80–200 cells in the IF measurement, *n* = 5 samples in the ELISA analysis). Repeated measures ANOVA followed by Dunnett’s post-test was used in the statistical analysis, and a *t*-test was also applied when the difference was not significant with the ANOVA test. The differences are considered statistically significant at *p* < 0.05. (P: plastic, G: hydrogel; line: line on the top of the box points to the compared objects with ANOVA, dot line: test with *t*-test).

To clarify how the AITC treatment affects the inflammatory response of CHs, we stained IL-6 and IL-10 in the CHs of each condition; we did so separately because of the same species of antibodies. We also stained the F-actin and nucleus at the same time and only presented their merged images to simplify the size of (Figure 7. The result showed that IL-6 and IL-10 (Figures 7A, B) levels were both increased from days 1 to 6 on plastic and hydrogel and were enhanced by AITC stimuli. Compared to growth on the plastic surface, the IL-6 and IL-10 levels were always higher on the hydrogel. Therefore, the inflammatory and anti-inflammatory responses were both promoted by AITC treatment. However, we speculated that the upregulated IL-6 might be triggered by some involved chemical component and not a result of the soft substrate; this hypothesis needs to be confirmed in further experiments.

Additionally, the ELISA method was also applied to test the cellular IL-6 and IL-10 contents. Under AITC treatment, the higher IL-6 level suggested an increased inflammatory response, and the higher IL-10 level suggested an increased anti-inflammatory response. However, only the IL-6 level was increased over time, and a soft substrate only enhanced the IL-10 expression. Collectively, the data indicated that the AITC supported a stronger anti-inflammatory action, which was much more significant on the soft matrix.

## Discussion

Since 1999, TRPA1 has been described as a non-selective cation widely expressed in sensory neurons, mediating nociception and neurogenic inflammation (Jaquemar et al., 1999; Koltzenburg, 2004). TRPA1 has been a more frequently researched TRP channel family member in recent years, and its functions are persistently implicated in OA. In most arthritis studies, TRPA1 was reported to be increased and acted as a promoter of joint destruction, mediating inflammatory and catabolic effects (Nummenmaa et al., 2016), oxidative stress (Phull et al., 2018), apoptosis (Yin et al., 2018), edema, and pain (Garrison and Stucky, 2014). However, how TRPA1 influences the pathogenesis of OA and CH dedifferentiation is less clear. As an inherent mechanosensitive ion channel, TRPA1 can be triggered by a variety of stimuli, such as temperature, electrophilic chemicals, and mechanical force (Corey et al., 2004; Moparthi and Zygmunt, 2020). With the development of OA, a hallmark of change is the thinner and stiffer cartilage layer, resulting from the altered synthesis of the collagen types with a lower collagen Ⅱ and higher collagen Ⅰ expression (Verzijl et al., 2002; Maldonado and Nam, 2013). It is well-known that matrix stiffness is involved in OA-caused changes in CH phenotype (Li et al., 2013). Thus, it would be beneficial to clarify whether substrate stiffness influences the TRPA1 activity and how CHs respond to TRPA1 activation in an environment of different stiffness.

In the present study, we again confirmed the upregulated TRPA1 expression of both protein and mRNA levels in OA cartilage, but it was not significantly higher than that of the normal cartilage. Thus, it might be the activation but not the overexpression that significantly affects the functions of CHs and cartilage. To reveal the response of CHs to the TRPA1 activation on different matrix stiffness, we used the normal plastic plate as the stiff substrate and PAA hydrogel as the soft substrate to culture CHs and activate the TRPA1 channel. Although acrylamide is harmless for animals, only high-level exposure (0.2 μg/kg/bw/day) can lead to a neurotoxic effect on humans (Exon, 2006). Therefore, natural PAA hydrogel is safe for cell culture and human use. After the 6-day monolayer culture, the TRPA1 expression was both upregulated but not different on a soft and stiff substrate. As a non-selective cation channel, TRPA1 can permeate Ca^2+^, Na^+^, and K^+^, and its activation leads to a substantial Ca^2+^ influx into the stimulated cells (Nilius, 2007; Rosendahl et al., 2016). Here, AITC-caused TRPA1 channel activation in CHs was verified by measuring the Ca^2+^ influx. However, we observed the AITC treatment only activated the TRPA1 but did not promote its expression, indicating that all the effects influenced by AITC on CHs were from the functionality but not the amount of TRPA1.

To fully understand the effects of TRPA1 on the phenotype of CHs in OA, the cell shape, F-actin cytoskeleton, focal adhesion, METF-A and pSOX9 expression, collagen synthesis profiles, and inflammation-related actions were analyzed. As a sensitive dedifferentiation characteristic of CH, the changes in cell shape were easily noticed during cell growth. Long-term culturing resulted in a hypertrophic and fibrotic morphology of CHs, which can be prevented by a soft culture environment, as previously reported (Schuh et al., 2012; Kim et al., 2015). Interestingly, the AITC treatment further increased the area and decreased the circularity of CHs on a stiff substrate, but it increased the circularity and did not affect the cell area on a soft surface. As known, actin polymerization is tightly connected to CH shape and phenotype. The intracellular actin in CHs can be found as a globular monomeric form called G-actin or a filamentous form called F-actin. During CH dedifferentiation, the G-actin monomers will polymerize as multimers (F-actin), whereas F-actin can also depolymerize back to G-actin, which is regulated by some actin-depolymerizing factors (Pollard and Cooper, 2009; Dominguez and Holmes, 2011). A healthy CH presents punctate actin in the cytoplasm, low F-actin, and a small spreading area, whereas dedifferentiated fibroblastic CH has a high F-actin and large spreading area (Shin et al., 2016; Parreno et al., 2017).

In this study, the total amount of F-actin was not changed during the 6 days of culture, but it was significantly increased under AITC treatment, which could be the result of the upregulated vinculin expression. Vinculin plays a role in controlling cell shape, where it bonds transmembrane proteins to the actin cytoskeleton (Tolbert et al., 2013). Additionally, the actin polymerization was much prevented on a softer surface even when the TRPA1 was activated. Therefore, AITC promoted vinculin and F-actin formation, and CHs presented a much more dedifferentiated phenotype on a stiff surface than on the soft one.

Collagen Ⅱ is the most important protein that CHs secrete for maintaining ECM stability. With OA development, less type Ⅱ but more type Ⅰ collagen is produced as one phenotypic characteristic of dedifferentiated CH (Charlier et al., 2019). On the stiff surface, collagen Ⅱ and Ⅰ were both increased after 6 days of culture, and the AITC further promoted the synthesis of these two proteins. Interestingly, CHs secreted more collagen Ⅱ but not collagen Ⅰ on the soft surface, which was positive for the redifferentiation of CHs. In addition, the AITC did not enhance the collagen Ⅰ but only collagen Ⅱ expression on the soft substrate. Thus, on stiff surfaces, AITC can have positive and negative effects on the collagen synthesis profiles of CHs, but on soft surfaces, its activation can specifically promote collagen Ⅱ production.

Because transcription factors MRTF-A and pSOX9 are, respectively, responsible for mediating the secretion of collagen Ⅰ and Ⅱ (Parreno et al., 2014; Lefebvre et al., 2019), we also analyzed the MRTF-A and pSOX9 contents in the total cell and nucleus. The phosphorylated SOX9 transfers into the nucleus and binds to essential sequences for coding the production of cartilage-specific ECM, especially collagen Ⅱ (Huang et al., 2000). In addition, MRTF-A is a G-actin binding protein, and the G-actin polymerization in dedifferentiated CHs can lead to MRTF-A nuclear localization and promote collagen Ⅰ expression (Parreno et al., 2014). The data in our study suggested that a softer matrix resisted the nuclear location of MRTF-A and also prevented the nuclear loss of pSOX9. AITC promoted both cellular and nuclear MRTF-A and pSOX9 contents on a stiff surface. However, on the soft substrate, AITC only caused a lower level increase of nuclear MRTF-A location and a more significant increase of pSOX9 expression in both the total and nucleus regions, indicating that the matrix stiffness determines the final effects of AITC treatment on MRTF-A and pSOX9 expression.

TRPA1 activation is a mediator in inflammatory action during cartilage degradation and joint pain (Moilanen et al., 2015b; Chow and Chin, 2020), specifically driving the IL-6 expression as a major player in the pathogenesis of OA (Nummenmaa et al., 2020). As previously reported, we also observed the increased IL-6 expression triggered by the AITC treatment. However, CHs expressed a higher level of IL-6 on the soft hydrogel surface than on the stiff plastic, which could be induced by the chemical composition of the hydrogel and not related to TRPA1 activation. More evidence should be verified in future studies. Apart from the proinflammatory effect, no one has claimed that TRPA1 is also related to the anti-inflammatory process. In the present study, we measured one anti-inflammation-related IL-10 expression (Miller et al., 2014; Li et al., 2022) and found that AITC enhanced IL-10 production, especially on a soft substrate. Thus, as far as we are aware, for the first time, it was found that AITC treatment or possible TRPA1 activation has both pro- and anti-inflammatory functions in the CH dedifferentiation process, and a soft environment may strengthen them all.

The limitations of this study are as follows: 1) we only used the AITC to imitate the activation of TRPA1 in OA, but the dysfunction of TRPA1 was not tested and discussed, which would not cover the full mechanism of how TRPA1 influences the CH phenotype and OA process; 2) the AITC was also reported to have antioxidant (Caglayan et al., 2019) and bactericidal activity (Lin et al., 2000), which were not tested but may also protect the CH phenotype; 3) the methodological approach was not diversified. Therefore, in further studies, we would apply TRPA1 inhibitors or siRNA to further clarify how the downregulation of TRPA1 affects the phenotype of CH and use more alternative methods to clarify whether it is also matrix stiffness-associated.

## Conclusion

**FIGURE 8:**
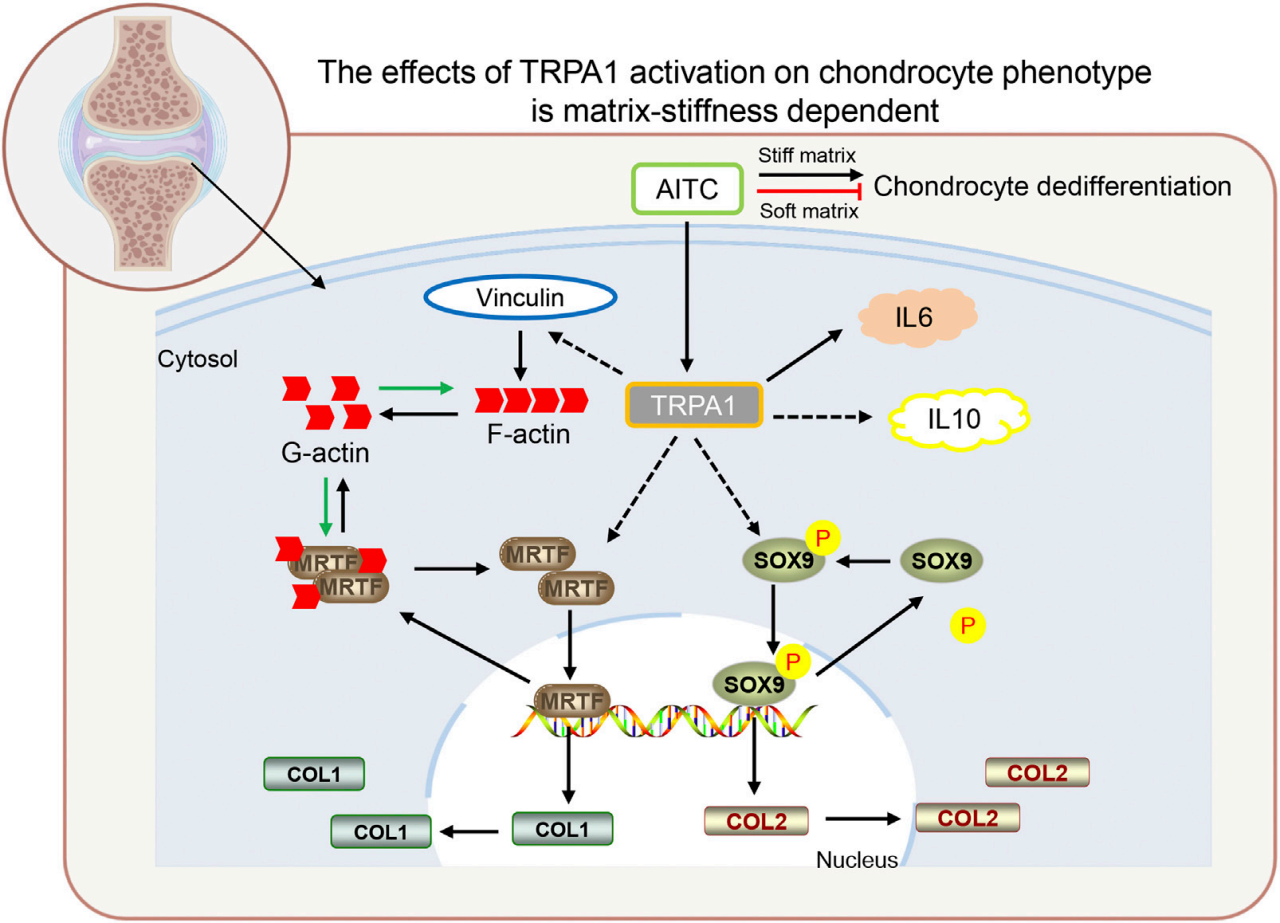
Proposed model depicting the TRPA1 activation in regulating CH dedifferentiation during OA. In general, TRPA1 activation promotes CH dedifferentiation on a stiff matrix but prevents CH dedifferentiation on a soft matrix. The AITC induces TRPA1 activation, which triggers vinculin upregulation and follows an increased F-actin expression. TRPA1 activation also induces the MRTF-A and pSOX9 upregulation, which transfers into the nucleus and codes collagen Ⅰ and Ⅱ expression. In addition, TRPA1 activation can increase both IL-6 and IL-10 expression. (The relations indicated by the solid lines are those verified in previous studies and those indicated by the dashed lines are newly identified in this study).

In general, the AITC treatment causes TRPA1 activation and acts as a double-edged sword that brings both positive and harmful effects on the chondrogenic phenotype of CHs. The positive effects are increased collagen Ⅱ, pSOX9, and IL-10 expression. The harmful effects are a much big cell spreading area, less circularity, more collagen Ⅰ, MRTF-A, F-actin, vinculin, and IL-6 expression (Figure 8). However, the harmful effects would be alleviated or resisted and the positive effects would be enhanced in a softer matrix environment. In detail, AITC treatment or potentially TRPA1 activation on a soft matrix results in a round morphology, much more collagen Ⅱ, pSOX9, and IL-10 expression. Meanwhile, it also causes decreased nuclear MRTF-A, F-actin, and vinculin but higher IL-6 expression. Therefore, how CHs respond to AITC treatment or potentially TRPA1 activation is determined by the cell feeling matrix stiffness. These findings may provide new insights into understanding the mechanisms of how TRPA1 influences the OA process and how interventions can be guided in positive and healthy directions.

## Data Availability

The original contributions presented in the study are included in the article/Supplementary Materials; further inquiries can be directed to the corresponding authors.
